# Psychometric validation of teacher empathy scale: Measurement invariance in gender

**DOI:** 10.3389/fpsyg.2022.1042993

**Published:** 2022-11-25

**Authors:** Abdolvahab Samavi, Kobra Hajializadeh, Moosa Javdan, Mohamad Reza Farshad

**Affiliations:** ^1^Department of Educational Sciences, University of Hormozgan, Bandar Abbas, Iran; ^2^Department of Psychology, Islamic Azad University, Bandar Abbas, Bandar Abbas, Iran; ^3^Department of Counseling, University of Hormozgan, Bandar Abbas, Iran

**Keywords:** teachers empathy scale, cognitive empathy, affective empathy, measurement invariance, psychometric validation

## Abstract

Result from Wang et al. study described the development and validation of an empathy scale for teachers (EST) and suggested that the EST could be an effective tool to assess the empathy of primary, middle and high school teachers in relation to their students. This study examines the factorial structure and factorial invariance of the EST in an Iranian sample. Confirmatory factor analysis was conducted to explore dimensionality and test for measurement invariance in factor structure, factor loadings and intercepts across gender in a sample (N = 462), of Iranian high-school male and female teachers (24–55 years). The data supported the multidimensional structure in both male and female samples. Accordingly, all factor loadings were significant and scale structure confirmed like the original scale. The results indicated that the EST includes three dimensions: cognitive empathy, negative affective empathy and positive affective empathy, and the internal consistency reliability of the three subscales are satisfactory in total sample and both sexes. Furthermore, the results revealed that invariance of the measure according to gender was confirmed. In addition, as the validity evidence, the EST is positively correlated with empathic concern scale scores. The study suggests that the EST could be an effective tool to measure the empathy of high school teachers in relation to their students in Iranian sample.

## Introduction

Background: Empathy is the ability to emotionally recognize what other people feel, see world from their point of view, and imagine yourself in their place. Empathy was defined by Baron-Cohen and Wheelwright (2004) as the drive to recognize another individual’s thoughts and emotions following through with proper emotional responses. The capability at understanding others’ experiences is called empathy, which reflects that comprehension of them (Hojat et al., 2002). Complex perspectives, emotions, and reactions may be included in others’ experiences, that is not visible externally existing within an individual’s life (Okun et al., 2000). For personality and social psychology, the concept of empathy possesses central significance, along with several other domains such as clinical/abnormal psychology, neuroscience, the health professions, and medicine (Hall and Schwartz, 2019). Empathy was introduced as a fundamental factor of the maintenance and development of close interpersonal relations (Coutinho et al., 2014) as well as a key motivational resource for prosocial performance (Okun et al., 2000).

The word “empathy” was appeared in 1908 as a translation of the German Einfühlung (literally “in-feeling”) with an aesthetic empathy meaning that American psychologists began to extend its scope to include the understanding of other people (Susann, 2018). By the Second World War, social psychologists began formulating examinations to measure a subject’s empathy for others (Susann, 2018). Earlier theorists claim that our ability to see movement and feeling in the world around us clarifies our capability to display and to experience beauty and kindness that were central to the construct of empathy (Gabrielle Starr, 2013).

Empathy has been extensively highlighted in the field of education, in teacher-student relationships (Williams et al., 2015; Wang et al., 2022). Recently, the educational psychologists focused on the factors of effective learning and teaching. Empathy is a key feature of teachers enabling acceptable communication between the pupils and their teachers. This ability requires emotional competencies to successfully perform different professional roles of teachers. Empathy is a dominant device that can help teachers better understand what’s driving the students’ behavior and find strategies to help them. According to earlier studies higher levels of empathy make people more productive in cooperative learning and work environments, and empathy in education setting has boost effects on academic success as well (Barr, 2011).

Teacher empathy is the teachers’ ability to comprehend and share the emotional states of the students in the educational context. Thus, the student is the empathized and the teacher is the empathizer. Only the attribution of students’ emotions is included in teacher empathy with no mental states. Moreover, a feeling of pity for the pupil’s misfortune and pain is included (Wang et al., 2022). The role of empathy in successful teaching has been supported in different studies (McAllister and Irvine, 2002; Arghode et al., 2013; Murphy et al., 2018). It has been found that the empathic ability is related to the teachers’ self–efficacy (Goroshit and Hen, 2016), school culture perceptions (Barr, 2011), teachers interaction and caring (Cooper, 2004), science education (Arghode et al., 2013), and the teachers’ emotional self-efficacy (Goroshit and Hen, 2014).

Regarding the general concept of empathy, several researchers have accepted empathy as a multidimensional structure while recognizing both affective and cognitive components of empathy. Cognitive empathy represents the capability to comprehend another individual’s emotional state. However, affective empathy denotes the ability to vicariously share or experience another individual’s emotional state (Preston and De Waal, 2002; Baron-Cohen and Wheelwright, 2004). In educational practice, it is essential to identify and understand the feelings of students from their view point. It is also vital to engage oneself in the position of the student to experience his/her feelings for all professional work of every teacher (Wang et al., 2022). Studies have been also performed on the affective and cognitive components of empathy on teacher empathy (Zhu et al., 2019).

### Gender differences In empathy

According to the popular culture and common stereotypes, women have a higher capacity to understand feelings and thoughts of others than men (Hodges and Klein, 2001). Moreover, based on empirical evidence, there are some differences between female and male in empathy (Macaskill et al., 2002; Toussaint and Webb, 2005). Gender differences in empathy have been investigated in several studies. For instance, Toussaint and Webb (2005) examined gender differences in 127 community residents and indicated that women were more empathic than men. According to Trentini et al. (2022), girls represented greater emotional empathy than boys. Also, Benenson et al. (2021) showed that women have greater empathy than men. It was believed that these differences were somewhat explained by the emotional self-awareness. According to some studies, the women had higher empathy (especially emotional empathy) than men and attributed to factors like gender roles and hormones (oxytocin and testosterone) (Rueckert and Naybar, 2008; Chen et al., 2014). Considerable gender differences were found by Arango-Tobón et al. (2020) in empathy. They revealed that women represented significant scores for cognitive/emotional empathy. Females scored higher in the studies of Michalska et al. (2013) on self-reported dispositional empathy than males. This difference increased with age. Strekalova et al. (2019) represented more complex maps of female students also including larger levels and number of empathy-related concepts. On the other hand, in numerous studies, no considerable gender differences were reported in empathy-based and empathy constructs (Ickes et al., 2000; Thomas and Maio, 2008; Lamm et al., 2011; Rand et al., 2016; Löffler and Greitemeyer, 2021). Totally, the assessment method highly orients the sex differences in empathy. Higher empathy levels in women were found in self-reported empathy scales. However, in neurophysiological measures of empathy no sex-based differences were found. Thus, the present work aimed to discover gender differences in verbal responses of the participants to an empathy questionnaire to recognize these gender differences.

### Empathy measurement

To measure the empathy, some empathy scales have been developed including Balanced Empathy Quotient (Baron-Cohen and Wheelwright, 2004), Emotional Empathy Scale (Mehrabian, 1997), Basic Empathy Scale (Jolliffe and Farrington, 2006) and Toronto Empathy Questionnaire (Spreng et al., 2009), which were established in the general population. According to (Wang et al., 2022), empathy is highly dependent on the context. Moreover, teachers may have different empathy for their pupils in educational contexts randomly others. Therefore, these scales may have no predictive and explanatory value to assess the empathy of the teachers regarding their pupils.

Some efforts have been made so far to assess empathy in teachers. Barr (2011) studied the association between empathy of the teachers and perceptions of the culture of their school. Using the Interpersonal Reactivity Index (Davis, 1983), he assessed the empathy in teachers. In fact, no difference was found between teacher empathy and general empathy. Thus, no new tool was developed for measuring it. A scale was developed by Vorkapić and Ružić (2013) for measuring the teacher empathy as a multidimensional construct. It included four dimensions of fantasy, empathic concern perspective taking, and personal distress among future kindergarten teachers in terms of Interpersonal Reactivity Index (IRI). According to Vorkapić and Ružić (2013), all four scales possess satisfactory coefficients of internal reliability within the range of 0.66 to 0.83. This scale cannot be considered as a teacher’s empathy scale owing to developing IRI-based items despite the good reliability of the subscales.

A tool was developed and validated by Bouton and Buxton (2014) within the field of education for measuring teacher empathy for first time. It included a 21 item self-report scale for assessing empathy in secondary school teachers. The validity and reliability of this scale was confirmed by Bouton and Buxton (2014). However, Wang et al. (2022) criticized some items of this scale associated with other constructs like prosocial behavioral tendencies rather than empathy. Recently, an empathy scale was developed and validated Wang et al. (2022) for teachers (EST) as an educational context construct in terms of an open ended survey of teachers. As we know, this is the most recent scale for measuring teacher’s empathy. Concerning the Iranian society, we found that there is no independent scale of empathy for teachers in Iran. Moreover, for assessment empathy in teachers, the general questionnaires of empathy such as empathy quotient (Baron-Cohen and Wheelwright, 2004) and the multidimensional emotional empathy scale (MDEES) (Caruso and Mayer, 1998) were utilized.

Accordingly, we assessed the psychometric features of empathy scale for teachers (EST) (Wang et al., 2022) in the present study. For this purpose, we tried to validate an educational context scale of empathy. EST is a well-developed scale with good reliability and validity. Thus, we assessed its validity through a confirmatory factor analysis. We also assessed its association with empathic concern scale scores as the convergent validity evidence. Then, we explored the differential functioning of the items and the invariability of this scale based on the gender concerning the gender differences in empathy, mainly the confirmed gender differences in self-reported empathy scales.

## Materials and methods

### Participants

462 high school teachers participated in this study, 230 (49.80%) of whom were men and 232 (50.20%) were women, with age ranges from 24 to 55 (M = 33.34; SD = 7.51). They were all random selected *via* an online survey website. We selected the participants from the different states of country to have a representative sample of the high school teacher population. The participants reported their demographic information, including age, sex, and years of teaching. Besides, an informed consent form was filled by the participants before they completed the questionnaires. The confidentiality of data and its application only for scientific purposes was informed to the participants and it was guaranteed that personal data will be kept anonymous. Participants’ demographic data is presented in Table 1.

**Table 1:** Description of participants’ demographic variables.

	N (%)	Mean	SD
Sex			
Female	232(50.20%)		
Male	230(49.80%)		
Age		33.34	7.51
24–34	156(33.76%) (male = 77, female = 79)		
35–44	165(35.72%) (male = 82, female = 83)		
45–55	141(30.52%) (male = 71, female = 70)		
Level of education completed			
Bachelor Degree	198(42.85%)		
Master’s Degree	189(40.91%)		
Doctorate Degree (PhD)	75(16.24%)		
	Total 462 (100)		

### Instruments

Using demographic data sheets, data on age, educational level, and sex was collected. The empathy of participants was measured using empathy scale for teachers (EST). EST assesses empathy using 19 Likert-scale items (1 = completely disagree; 4 = completely agree). Its original English version covers three factors: (a) Cognitive empathy, involving inferring, recognizing, and understanding emotions of students (items 1–9) (sample item: I can quickly tell whether my pupils are happy or not), (b) Negative affective empathy, including experiencing and sharing negative emotions of students (items 10–14) (sample item: I sometimes get caught in the negative emotions of my pupils), and (c) Positive affective empathy, involving positive emotions as well (items 15–19) (sample item: Seeing the pupils happy makes me very happy). By summation of the item scores, the scores on the EST subscales can be calculated. The total empathy score is obtained by the sum of three subscales. Wang et al. (2022) used confirmatory factor analysis, exploratory factor analysis, and convergent validity in order to investigate the validity of this scale and presented satisfactory evidence. These authors also reported acceptable reliability coefficients for this scale (EST total score, alpha = 0.81; negative affective empathy, alpha = 0.74; cognitive empathy, alpha = 0.84; positive affective empathy, alpha = 0.78).

The empathic concern subscale: We implemented the Persian version of the empathic concern subscale of the Interpersonal Reactivity Index questionnaire (Davis, 1983). It evaluates whether the person has the tendency to experience empathetic feelings toward those in distress, and it is supposed to assess empathy. This subscale includes 7 items and it is scored based on a 5-point Likert scale ranging from 1 (Does not describe me well) to 5 (Does describe me well). The reliability and validity of IRI and this subscale was reported as satisfactory by Davis (1983). Its reliability for the Persian version has been reported as 0.69 (Khodabakhsh and Mansori, 2012). In our work, Cronbach’s alpha for the subscale was 0.73.

### Procedure

In order to investigate the psychometric characteristics of EST, its original version was translated to Persian using a standardized translation process (Gjersing et al., 2010). EST was translated into Persian by two external translators and two bilingual experts (English-Persian) based on the International Test Commission’s rules (Hernández et al., 2020). A bilingual Doctor of Psychology revised the translations and translated them back into English (Gudmundsson, 2009). He was not related to this study and provided necessary terminological adjustments in some terms not agreed upon by the previous translators. Lastly, data was collected through an online survey that was distributed through mobile media and social networks. The informed consent form and the questionnaires were completed in Persian by the participants.

### Data analysis

SPSS-26 software was used for descriptive analysis of data, and then a confirmatory factor analysis (CFA) was done with the original 3D version scale by the use of Amos 24.0. The fit indices used the approximation mean square error (RMSEA), the ratio χ2/df, and the comparative fit index (CFI). By the RMSEA approaching 0.06, the CFI ≥ 0.90, and the ratio χ2/df > 3, the goodness-of fit model was regarded as satisfactory. The RMSEA with 90% CI, Δχ2 and ΔCFI were employed for the invariance of the measure as an incremental adjustment index. With the *p* > 0.05 of Δχ2 (given the sample size bias), there is invariance of the measure; the RMSEA values ≤0.05 and the ΔCFI value of the models compared is <0.01 (Byrne, 2016). With conducting an analysis of configural invariance, it was tested whether both groups showed the same number of factors and pattern of loadings. Besides, metric invariance was used for checking whether each item has a contribution to the latent construct to a similar degree across groups. Metric invariance is investigated by making constraint on factor loadings (that is, the item loadings on the constructs) being equivalent in both groups. There is a scalar invariance when mean differences in the latent construct capture all mean differences in the shared variance of the items. By constraining the item intercepts equivalent in the two groups, we tested scalar invariance. In order to examine the EST convergent validity, Pearson’s correlation coefficient was calculated with empathic concern subscale scores, and the internal consistency procedure was used to examine the reliability (Cronbach’s alpha coefficient). AMOS-24 and SPSS-26 were used to perform all analyses, and the statistical significance level was a minimum of *p* < 0.05 in all analyses.

## Results

### Descriptive results

Table 2 presents the mean of scores of the EST items in the total sample. It also provides the item reliability and normality indices.

**Table 2:** Descriptive statistics, normality indices, and item analysis of Empathy Scale for Teachers (EST) (n = 462).

	Mean (SD)	r item-total	Skewness	Kurtosis	K-S	*α if item deleted*
			SE(0.227)	SE(0.114)		
Item 1	2.87 (0.90)	0.68^**^	−0.48	−0.52	0.25	0.93
Item 2	2.88 (0.95)	0.76^**^	−0.50	−0.65	0.24	0.93
Item 3	2.55 (1.01)	0.65^**^	−0.08	−1.08	0.20	0.93
Item 4	2.80 (0.91)	0.67^**^	−0.27	−0.79	0.22	0.93
Item 5	2.67 (0.99)	0.80^**^	−0.31	−0.92	0.24	0.93
Item 6	2.64 (0.91)	0.74^**^	−0.14	−0.79	0.22	0.93
Item 7	2.75 (0.92)	0.73^**^	−0.32	−0.72	0.24	0.93
Item 8	2.65 (0.98)	0.67^**^	−0.15	−0.99	0.20	0.93
Item 9	2.55 (0.97)	0.65^**^	0.021	−1.01	0.21	0.93
Item 10	2.75 (0.87)	0.61^**^	−0.25	−0.61	0.24	0.93
Item 11	2.80 (1.01)	0.70^**^	−0.42	−0.91	0.23	0.93
Item 12	2.70 (1.02)	0.60^**^	−0.24	−1.07	0.20	0.93
Item 13	2.76 (0.97)	0.69^**^	−0.28	−0.93	0.21	0.93
Item 14	2.60 (0.97)	0.64^**^	−0.12	−0.96	0.20	0.93
Item 15	3.06 (1.04)	0.67^**^	−0.76	−0.59	0.26	0.93
Item 16	2.87 (1.03)	0.64^**^	−0.49	−0.91	0.22	0.93
Item 17	2.52 (0.97)	0.69^**^	−0.13	−0.985	0.23	0.93
Item 18	2.87 (1.04)	0.70^**^	−0.52	−0.941	0.22	0.93
Item 19	2.91 (1.04)	0.64^**^	−0.61	−0.806	0.23	0.93
Cognitive empathy	24.39 (6.58)	0.92^**^	−0.42	−0.62	0.09	0.91
Negative affective empathy	13.63 (3.90)	0.81^**^	−0.38	−0.65	0.11	0.86
Positive affective empathy	14.25 (4.15)	0.82^**^	−0.67	−0.45	0.13	0.87
Total	52.27 (12.69)	1	−0.45	−0.323	0.06	0.93
Empathic concern	17.47 (6.63)	0.34^**^	0.63	−0.41	0.10	0.73

** Significant correlation at the 0.01 level (2-tailed).

### Confirmatory factor analysis (CFA) results

The factor structure of EST was investigated using a CFA with maximum likelihood estimation. The model fit was examined using the CFI, the RMSEA, and the chi-square to degree-of-freedom ratio (χ2/df). There are threshold values of the fit indices that we compared the calculated value to them. The CFI values above 0.90, RMSEA values below 0.08, and χ2/df values below 3.0 (or 5.0) showed a good fit (Schermelleh-Engel et al., 2003). Our findings showed the significance of all factor loadings and scale structure was proved like the original scale. The results indicated that EST is composed of three constituents: positive affective empathy, cognitive empathy, and negative affective empathy in total sample and both genders. Table 3 shows the model fit indexes of total, male and female samples. As observed in this table, the model has an acceptable fit for the data in total, male, and female samples. Table 4 presents the factor loadings of the scale items in total, male, and female samples. It is observed that all factor loadings are larger than 0.30 and significant.

**Table 3:** Model fit indices of total, female and male samples.

	χ2	df	χ2/df	p	RMSEA (CI 95%)	CFI
Women	296.88	147	2.02	0.001	0.06[0.055; 0.077]	0.95
Men	252.13	149	1.69	0.001	0.05[0.043; 0.067]	0.95
Total	530.080	149	3.55	0.001	0.07[0.068; 0.081]	0.92

**Table 4:** Factor loadings of the scale items in the in total, female and male samples.

		Total		Women		Men	
		Beta	p	Beta	p	Beta	p
Cognitive empathy	Item 1	0.71	0.001	0.79	0.001	0.65	0.001
	Item 2	0.78	0.001	0.80	0.001	0.75	0.001
	Item 3	0.67	0.001	0.72	0.001	0.62	0.001
	Item 4	0.68	0.001	0.72	0.001	0.64	0.001
	Item 5	0.82	0.001	0.83	0.001	0.81	0.001
	Item 6	0.75	0.001	0.76	0.001	0.74	0.001
	Item 7	0.75	0.001	0.77	0.001	0.74	0.001
	Item 8	0.71	0.001	0.78	0.001	0.64	0.001
	Item 9	0.67	0.001	0.73	0.001	0.61	0.001
Negative affective empathy	Item 10	0.75	0.001	0.78	0.001	0.71	0.001
	Item 11	0.80	0.001	0.88	0.001	0.73	0.001
	Item 12	0.68	0.001	0.73	0.001	0.62	0.001
	Item 13	0.71	0.001	0.78	0.001	0.64	0.001
	Item 14	0.78	0.001	0.82	0.001	0.74	0.001
Positive affective empathy	Item 15	0.76	0.001	0.79	0.001	0.72	0.001
	Item 16	0.71	0.001	0.78	0.001	0.64	0.001
	Item 17	0.74	0.001	0.78	0.001	0.70	0.001
	Item 18	0.80	0.001	0.85	0.001	0.75	0.001
	Item 19	0.77	0.001	0.83	0.001	0.70	0.001

### Measurement invariance

Table 5 presents the measurement invariance results. It is seen that CFA models determined for females and males showed an acceptable fit to the data, demonstrating that a multiple group CFA was appropriate. Before the testing the invariance between sexes, an independent samples t-test was run (t value = 4.68, *p* < 0.01). The configural metric and scalar invariances were also examined. According to the results, there was a strong invariance between sexes. Table 5 shows that the increase in χ2 from the base model to the metric invariance model was 6.38 (Δχ2 = 6.38 (Δdf = 16); *p* > 0.05). Besides, the increase in CFI was 0.001 below the 01 criterion (Van de Schoot et al., 2012). Thus, the indexes approve scalar invariance between males and females. It is observed that the increase in χ2 from the metric invariance model to scalar invariance model was 27.19 (Δχ2 = 27.19 (Δdf = 19); p > 0.05) and the increase in CFI was 0.001 below the 0.01 criterion.

**Table 5:** Fit indices for the invariance test in gender groups.

	χ2	df	p	RMSEA (CI 95%)	CFI	Δχ2	Δ CFI
Women	440.29	149	0.001	0.08[0.072; 0.092]	0.91		
Men	252.13	149	0.001	0.05[0.043; 0.067]	0.95		
Configural invariance gender	692.16	298	0.98	0.054[0.048; 0.059]	0.924		
Metric invariance gender	698.55	314	0.53	0.052[0.046; 0.057]	0.925	6.38^ns^(Δdf = 16)	0.001
Scalar invariance gender	725.75	333	0.10	0.051[0.046; 0.056]	0.924	27.19^ns^(Δdf = 19)	0.001

### Convergent validity and reliability

The reliability of the EST and its dimensions was checked using Cronbach’s alpha. The correlation between EST scores and empathic concern scores was investigated for examining the convergent validity. There was a significant association between the total empathy score and the EST subscales with empathic concern scores. Table 6 indicates the correlation coefficients and Cronbach’s alpha.

**Table 6:** EST convergent validity and internal consistency reliability (n = 462).

	*α*	1	2	3	4	5
1. Total empathy	0.93	1				
2. Cognitive empathy	0.91	0.928^**^	1			
3. Negative affective empathy	0.86	0.812^**^	0.643^**^	1		
4. Positive affective empathy	0.87	0.824^**^	648^**^	0.523^**^	1	
5. Empathic concern	0.73	0.343^**^	0.298^**^	0.183^**^	0.405^**^	1

** Correlation is significant at the 0.01 level (2-tailed).

## Discussion

The present research aims at evaluating the psychometric features of EST in high school teachers in Iran, and its structural properties are explored and the suitable structure in the mentioned sample is confirmed. Besides, the factorial invariance is assessed based on gender, and the relationship with a convergent validity source (empathic concern) was evaluated. Research findings confirmed the EST reliability and validity. The results showed that the CFA is supporting a 3D structure of EST, which includes negative affective empathy, positive affective empathy, and cognitive empathy. It is in line with the present perspective of empathy, claiming empathy as a multidimensional construct that is composed of affective and cognitive dimensions (Davis, 1983; Cohen and Strayer, 1996; Baron-Cohen and Wheelwright, 2004; de Waal and Preston, 2017). Cognitive empathy is defined as accurately perceiving the emotional state of another, while affective empathy means the mediated affective response with the same emotion to the emotional state of another (Zaki and Ochsner, 2012). According to recent research, it is a common interpersonal phenomenon to share another one’s positive emotions, known as positive empathy, which enhances interpersonal outcomes, encouraging prosocial behaviors (Morelli et al., 2015). Previous research works have also indicated that feeling along with negative emotion of others is a capability distinctive from feeling along with positive emotions of others (Andreychik and Migliaccio, 2015; Andreychik and Lewis, 2017). Wang et al. (2022) stated that it is necessary and logical to separate negative affective empathy and positive affective empathy in the EST. Positive empathy comprises recalling, imagining, learning, or observing positive outcomes of others that can activate positive empathy. One may experience positive empathy as an uninvolved observer when they interact with others or when create a positive experience for someone else. Positive empathy may happen in response to various social targets, including groups or individuals, fictional or real characters, and distant or close others. Positive empathy can be experienced as a stable personality trait or a transient emotional state (Morelli et al., 2015). Additionally, negative affective empathy contains sharing negative emotions of others, involving emotional costs since we often feel bad when we observe the suffering of others (Zaki, 2014). Despite the existence of an association between both negative empathy and positive empathy and a similar degree of feeling to help others in need, positive but not negative empathy is associated with “every day” prosocial behaviors intended explicitly to increase the positive emotions of others (Andreychik and Migliaccio, 2015). Thus, it could be detrimental to show excessive sensitivity to others’ suffering and bring about some negative complications, like burnout or fatigue (Gleichgerrcht and Decety, 2013). Consistently, teachers act toward both negative and positive emotional empathy in their school activities. Behaviors associated with positive empathy are observed more frequently than those associated with negative empathy. Seeing a teacher that experiences positive emotions with his students’ success is more likely than seeing a teacher who tries to share his feelings with a depressed student. In general, it is necessary to make a distinction between the two types of empathy, and practically, it would be helpful for teachers in doing both types of empathy.

Moreover, Cronbach’s alpha was calculated for testing the EST reliability. As shown by the results, the whole scale and three subscales showed a high alpha coefficient, supporting reliability of the EST. Furthermore, convergent validity was assessed through the calculation of the EST correlation with empathic concern. According to the results, there is a significant correlation between the empathetic concern and the entire scale, positive affective empathy, negative affective empathy and cognitive empathy. The validity and reliability of the EST are evidenced by the analyses in our work.

The factorial invariance of the EST was examined in terms of gender. The measurement invariance results indicated that CFA models determined for females and males had an acceptable fit to the data. It shows that a multiple group CFA was suitable. The configural, metric and scalar invariances on gender indicate that both females and males perceive the EST items identically, which reveals good adjustment levels. The findings in our work show a good agreement with previous studies that did not find any gender differences in empathy (Lamm et al., 2011; Löffler and Greitemeyer, 2021). As already mentioned, there is not agreement in studies on gender differences in empathy. Nevertheless, the absence of any difference between empathy of males and females has been supported by most previous studies (Lamm et al., 2011; Rand et al., 2016; Löffler and Greitemeyer, 2021). Moreover, the studies have attributed the differences in empathy to the type of assessment approaches. That is, the measurement invariance of the EST in our work proved both the lack of significant difference in empathy between males and females and the lack of dependence of the participants’ answers on the empathy assessment method in a self-report scale.

### Implications

Significant implications are included in the present study for practitioners and scholars in the field of educational psychology since it presents a simply administered, psychometrically sound measurement tool. With a reliable and valid measurement tool, it is possible to propose experimental research of teacher empathy. The researchers can use EST for detecting individual differences and discovering empathy developmental patterns among female and male high school teachers. Besides, they can apply the EST for investigating the way of relationship between teacher empathy and other educational constructs. Future studies are recommended to focus on investigating the relationship between teachers’ self-efficacy and teacher empathy or the relationship between teachers’ professional development and teacher empathy. Additionally, given the positive impacts of teacher empathy, development of interventions for improving empathy skills in teachers is suggested and the EST can be used for evaluating the efficacy of empathy interventions.

### Limitations

Despite offering valuable information, the present study also has some limitations. First, the research was carried out on high school teachers in Iran, and the findings generalization to teachers of other educational levels like the elementary schools teachers should be done cautiously. Second, the research data was collected virtually, and the subjects completed the online questionnaire voluntarily. Collecting data in this way can cause sample bias. The probability of participation of those with high levels of altruism and empathy might be more. In order to enhance the EST validity, an important step is to investigate its measurement invariance between offline and online data sets. Third, since the EST is a self-report questionnaire, the researcher should consider the bias possibility in the participants’ responses. Fourth, the existing evidence for the EST validity is confined to correlations with empathic concern scores as the convergent validity evidence. Using other validity analysis approaches, like discriminant validity in future works is suggested.

## Conclusion

It is essential to identify an assessment test of empathy in the Iranian teachers that is not a similar scale for assessing it in the Iranian educational context. The results of the present study showed that the EST possesses suitable psychometric characteristics for research and intervention purposes. The EST was translated into Persian and enjoys acceptable validity and reliability implemented on a representative sample of teachers. As far as we know, this is the first work attempting to evaluate the reliability and validity of this scale and confirms the structure in a sample of Iranian teachers. Besides, from an applied perspective, it is important to consider the invariance of the measure according to gender. The EST has reliability in evaluating empathy in males and females and presents an acceptable internal consistency and a significant positive relationship with other empathy scores.

## Data availability statement

The original contributions presented in the study are included in the article/supplementary material, further inquiries can be directed to the corresponding author.

## Ethics statement

The studies involving human participants were reviewed and approved by ethics committee of University of Hormozgan. The patients/participants provided their written informed consent to participate in this study.

## Author contributions

AS, KH, MJ, and MF contributed to the study conception and design, material preparation, data collection and analysis. All authors contributed to the article and approved the submitted version.

## Conflict of interest

The authors declare that the research was conducted in the absence of any commercial or financial relationships that could be construed as a potential conflict of interest.

## Publisher’s note

All claims expressed in this article are solely those of the authors and do not necessarily represent those of their affiliated organizations, or those of the publisher, the editors and the reviewers. Any product that may be evaluated in this article, or claim that may be made by its manufacturer, is not guaranteed or endorsed by the publisher.
